# Danqi soft caspule alleviates myocardial ischemia-reperfusion injury induced cardiomyocyte apoptosis by attenuating mitochondrial fission

**DOI:** 10.3389/fphar.2025.1526253

**Published:** 2025-03-12

**Authors:** Ye Yang, Cuiting Lin, Yan Wang, Yu Liu, Qiuxiong Chen, Shiyu Ma, Jin Ma

**Affiliations:** ^1^ The Second Clinical College of Guangzhou University of Chinese Medicine, The Second Affiliated Hospital of Guangzhou University of Chinese Medicine, Guangdong Provincial Hospital of Chinese Medicine, Guangzhou, China; ^2^ State Key Laboratory of Dampness Syndrome of Chinese Medicine, Guangzhou, China

**Keywords:** myocardial ischemia-reperfusion injury, oxidative stress, cell apoptosis, mitochondrial dynamics, Chinese botanical drug

## Abstract

**Background:**

Myocardial ischemia-reperfusion (I/R) injury which leads to continuously worsening ventricular remodeling and cardiac dysfunction in the chronic stage, is a significant contributor to the global prevalence of heart failure. Traditional Chinese herbal formulas have been shown to prevent myocardial I/R injury.

**Method:**

This study aims to investigate whether Danqi soft caspule (DQ), a classical traditional Chinese medicine (TCM) preparation, exerted the protective effects against myocardial I/R injury and explore the potential underlying mechanisms. A rat model of myocardial I/R and a cell model of H_2_O_2_ induced oxidative stress injury were established to assess the effects of DQ on cardiac injury, cardiomyocyte apoptosis, as well as mitochondrial structure and function.

**Result:**

DQ pre-treatment reduced both the proportion of infarct area and ischemic risk area and decreased cardiomyocyte apoptosis in myocardial I/R injury rats. In H_2_O_2_ induced cells, DQ was found to reduce cell apoptosis and lower oxidative stress levels. Furthermore, DQ inhibited mitochondrial fission, prevented alterations in mitochondrial membrane potential, and suppressed Cytochrome C release from the mitochondria, thereby preventing apoptosis. DQ has protective effects against I/R induced oxidative stress injury by reducing cardiomyocyte apoptosis through inhibition mitochondrial fission. Moreover, DQ could restore mitochondrial structure and function by suppressing the phosphorylation of Ca^2+^/calmodulin-dependent protein kinase II (CaMKII) and dynamin-related protein 1 (Drp-1).

**Conclusion:**

DQ inhibited I/R injury and cardiomyocyte apoptosis by reducing mitochondrial fission associated with suppressing the phosphorylation of CaMKII and Drp-1.

## 1 Introduction

Acute myocardial infarction (AMI) resulting from coronary vascular occlusion is one the most prevalent cardiovascular causes of mortality worldwide. In the early stages, timely reperfusion to the ischemic myocardium through thrombolytic therapy or percutaneous coronary intervention is the most effective treatment for AMI, as it reduces myocardial ischemic injury and limits infarct size. However, even when revascularization is performed in time, it can lead to additional damage to cardiomyocytes, a phenomenon commonly referred to as myocardial ischemia reperfusion (I/R) injury. Myocardial I/R injury manifests in structural damage, disturbance in electrical activity and myocardial dysfunction during the acute phase. This may progress to ventricular remodeling, cardiac dysfunction and ultimately death in the chronic phase (Basalay et al., 2020). Although conventional pharmacological interventions and clinical reperfusion therapies have been shown to enhance emergency survival rates and improve short-term prognoses, they remain limited in their ability to provide sustained improvements in cardiac function or reverse pathological progression (Liu et al., 2024). Currently, due to a lack of effective clinical interventions, significant challenges persist regarding cardiac reperfusion therapy.

Myocardial I/R injury involves complex mechanisms, including intracellular Ca^2+^ overload, oxidative stress, and heightened inflammation. Research has indicated that mitochondrial homeostasis is crucial in the pathophysiological processes underlying myocardial I/R injury (Xin et al., 2021). Extensive mitochondrial fission adversely affects both the function and structure of mitochondria, leading to reduction in mitochondrial membrane potential (MMP) and overproduction of reactive oxygen species (ROS), which induces oxidative stress and exacerbates cardiomyocyte damage through the mitochondria-dependent apoptosis pathway (Xin et al., 2021; Sygitowicz and Sitkiewicz, 2022). Dynamin-related protein 1 (Drp-1), a large GTPase, serves as the primary molecular mediator of mitochondrial fission by oligomerizes the guanosine triphosphate (GTP) to constrict the outer mitochondrial membrane (Sharp, 2015). The regulation of Drp-1 activity is primarily modulated by phosphorylation. Specifically, Ser616 phosphorylation facilitates the translocation of Drp-1 to the outer mitochondrial membrane (Zhang et al., 2024). Inhibition of Drp-1 using mdivi-1 demonstrates cardioprotective effects during myocardial I/R injury by preventing excessive mitochondrial fission and fragmentation in prediabetic rats (Maneechote et al., 2023).

Danqi soft capsule (DQ), which contains *Salvia miltiorrhiza Bunge* and *Panax notoginseng (Burk.) F. H. Chen,* is a clinically utilized Chinese botanical drug for treating cardio-cerebrovascular diseases (Zheng et al., 2013). Our previous studies have shown that DQ can mitigate the risk of atrial fibrillation and ventricular arrhythmia in post-myocardial infarction rats by improving arrhythmogenic substrates primarily through the inhibition of fibroblast function (Ma et al., 2019; Ma et al., 2022). Notably, DQ exhibits a protective effect during the chronic phase of myocardial infarction. However, the impact of DQ on cardiomyocytes during the acute phase of ischemic heart disease remains to be investigated. The principal active components derived from *Salvia miltiorrhiza Bunge* are salvianolic acids, while those from *Panax notoginseng (Burk.) F. H. Chen* are panax notoginseng saponins. Previous reports indicate that both salvianolic acids and panax notoginseng saponins can attenuate cardiomyocyte apoptosis via mitochondrial pathways (Zhou et al., 2018; Li et al., 2019). In this study, we established a myocardial I/R injury model as well as an H_2_O_2_-induced oxidative stress injury cell model to explore the potential mechanisms underlying DQ’s alleviation of myocardial I/R injury through reducing cardiomyocyte apoptosis via mitochondrial homeostasis. This investigation aims to provide a theoretical basis for the clinical application of DQ in treating myocardial ischemic diseases.

## 2 Materials and methods

### 2.1 Animal model of myocardial I/R injury and groups

All animal experiment procedures were conducted in accordance with the Guide for the Care and Use of Laboratory Animals established by the National Institutes of Health, as well as with national standards set forth by the People’s Republic of China regarding ethical review for laboratory animal welfare. All protocols related to animal experimentation received approval from the Animal Ethics Committee of Guangdong Provincial Hospital of Chinese Medicine (Number: 2019070). Male Sprague-Dawley (SD) rats weighing approximately 280–300 g (SPF grade) were procured from Beijing Vital River Laboratory Animal Technology Co., Ltd (Beijing, China), which were randomly assigned into four groups: sham operation group (Sham), I/R model group (I/R), low-dose DQ group (DQ-L), and high-dose DQ group (DQ-H). Rats in both DQ group received intragastric administration at doses of 0.6 g/kg for DQ-L or 1.2 g/kg for DQ-H daily for 5 days prior to surgery according to the previous study (Ma et al., 2019; Ma et al., 2022). The I/R model was induced through ligation of the left anterior descending (LAD) coronary artery. Following thoracotomy to expose the heart, a 7–0 silk suture was employed to ligate the LAD approximately 2–3 mm below the tip of the left atrial appendage. After a period of 30 minutes, the ligature was loosened to restore the blood supply, after which chest closure occurred once reperfusion had been successfully reinstated. Animals in the Sham group underwent identical surgical procedures without LAD ligation. All samples were collected 24 h after reperfusion.

### 2.2 Preparation process and quality control of DQ

DQ is composed of 50% *Panax notoginseng* (Burk.) F. H. Chen [Araliaceae, *Notoginseng radix et rhizome*] and 50% *Salvia miltiorrhiza Bunge* [Lamiaceae; Salviae miltiorrhizae radix et rhizoma]. All medicinal materials are sourced from authentic suppliers by China Meheco Great Wall Pharmaceutical Co., Ltd. (Beijing, China) and are identified according to thin-layer chromatography (TLC) as outlined in Appendix VI B of the 2005 edition of the Chinese Pharmacopoeia. Detailed information regarding these two botanical drugs can be found in Supplementary Table 1. The supplementary data provides a comprehensive description of the extraction process and ultra-high performance liquid chromatography (UPLC) analysis of DQ.

### 2.3 Tetrazolium blue chloride (TTC)-Evans blue staining

The TTC-Evans blue staining technique was utilized to assess myocardial injury induced by I/R. Following a reperfusion period of 24 h, animals were anesthetized, and thoracotomy was performed to expose the heart. After opening the abdominal cavity, a 4% Evans blue staining solution was administered by intravenous injection. Subsequently, the heart was quickly cut off and stored at −20°C. Starting from the apex, the heart was transversely sliced into five to six equal portions of uniformly thick slices. These slices were immersed in a 2% TTC solution dissolved in PBS at 37°C for 15 min before being fixed in paraformaldehyde solution at room temperature. Determine the ischemic risk area (AAR) by the absence of blue dye. The total left ventricular area (LV), AAR, and TTC-negative staining area representing infarcted myocardium were quantified using ImageJ software.

### 2.4 Hematoxylin-eosin (HE) staining

Heart specimens were fixed with paraformaldehyde, embedded in paraffin wax, sectioned into thin slices measuring 5 μm each, and mounted onto glass slides for HE staining procedures. Observations and imaging were conducted under an optical microscope (Olympus, Japan). The pathology score of each heart slices is as follows: (0) nil; (1) minimum (focal myocytes damage); (2) mild (small multifocal degeneration with slight degree of inflammatory process, the order of myocardial fibers is occasionally disordered); (3) moderate (extensive myofibrillar degeneration and/or diffuse inflammatory process, the cells are moderately damaged, Myocardial fibers are deformed in a wave shape, and the nucleus is shed); (4) severe (necrosis with diffuse inflammatory process: The cell is severely damaged, the nucleus shrinks, and myocardial necrosis is accompanied by diffuse inflammation). Four different visual fields were selected for each slide, and the average of the histopathological scores of the four visual fields was calculated to obtain the histopathological score of the slide, the analysis was conducted in a blinded way (Yin et al., 2020).

### 2.5 TUNEL staining

TUNEL assay kit (G1504, Servicebio) was used to detect the apoptosis of cardiac tissue. Paraffin-embedded heart tissue sections were prepared. After dewaxing and membrane breaking, DAPI staining, incubation and mounting were performed according to the kit instructions. Microscopic images were captured using an Olympus microscope (Japan). The apoptosis rate was calculated by ImageJ software to calculate the total number of apoptotic nuclei and nuclei in the field of view. Cardiomyocyte apoptosis rate = number of apoptotic nuclei/total number of nuclei × 100%.

### 2.6 Transmission electron microscope

The rat heart was fixed with electron microscope solution, and samples measuring approximately 1 mm^3^ were taken within 1–3 min. The myocardial tissue was immersed in the electron microscopy solution for fixation and stored at 4°C. Following a series of procedures including rinsing, fixing, dehydration, embedding, polymerization, positioning, and solidification, ultra-thin sections with a thickness of 60–80 nm were prepared. These sections were stained with a saturated alcohol solution of 2% uranium acetate for 8 min in darkness. After washing three times with 70% alcohol and ultrapure water, the sections were treated with a light-protected solution of 2.6% lead citrate for another 8 min. They were then washed three times with ultrapure water before being blotted dry on filter paper. Finally, the copper mesh slices were placed into a copper box and allowed to air-dry at room temperature overnight. The mitochondrial ultrastructure was observed using a Hitachi transmission electron microscope (Japan), and images were collected. The total number of mitochondria was counted by ImageJ software, and further divided it into three intervals according to the mitochondrial area: <0.5 μm^2^, 0.5–1 μm^2^, >1 μm^2^. The proportion of mitochondria in these three intervals was calculated, and the number of mitochondria in these three area intervals was statistically analyzed (Sun et al., 2022).

### 2.7 Cell culture and treatment

H9c2 cardiomyocytes, obtained from Meisen Chinese Tissue Culture Collections (China), were cultured in high-glucose Dulbecco’s Modified Eagle Medium (DMEM) supplemented with 10% fetal bovine serum. The cells were maintained in a humidity cell incubator at 37°C with 5% CO_2_. The culture medium was replaced every two to 3 days. The cardiomyocytes were divided into four experimental groups: control group (Con), H_2_O_2_ group (H_2_O_2_), low-dose DQ group (DQ-L) and high-dose DQ group (DQ-H). For cell experiments, the water-soluble components of DQ were utilized. Prior to modeling, the cardiomyocytes underwent pretreatment with DQ for a duration of 2.5 h.

### 2.8 Cell viability assay

Cell viability was assessed using the methyl thiazolyl tetrazolium (MTT) assay. Cells were seeded into 96-well plates at a density of 5 × 10^3^ cells per well and cultured at 37°C. Following various treatments as described above, the cells were washed twice with phosphate-buffered saline (PBS) before adding MTT (Sigma Aldrich,Unites States) to each well at a final concentration of 0.5 mg/mL for 4 h incubation. Subsequently, 100 μL of dimethyl sulfoxide (DMSO) was added to each well to dissolve the formazan crystals at room temperature for 10 min. The optical density (OD) value of each well was recorded at a wavelength of 570 nm using a microplate reader (Thermo Scientific, Unites States).

### 2.9 Flow cytometry

H9c2 cells were cultured in 6-well plates at a density of 2.5 × 10^5^ cells per well. Following treatment, the apoptosis rate was analyzed using the Annexin V-FITC apoptosis detection kit (Beyotime, China) according to the manufacturer’s instruction. The mitochondrial membrane potential was measured with JC-1 (Beyotime, China), which serves as a specific probe following the manufacturer’s protocol. The opening degree of the mitochondrial permeability transition pore (mPTP) was evaluated using a mPTP detection kit (Beyotime, China), employing Calcein AM as a fluorescent precursor compound according to manufacturer guidelines. Additionally, intracellular reactive oxygen species (ROS) levels were determined utilizing a ROS detection kit (Beyotime, China), adhering strictly to the manufacturer’s instructions. All fluorescence intensities were quantified with a flow cytometer (Agilent, Unites States).

### 2.10 Enzyme-linked immunosorbent assay (ELISA)

The activity levels of lactate dehydrogenase (LDH; Sigma-Aldrich, Unites States) and cardiac troponin T (cTnT; Elabscience, China) in serum were evaluated according to the protocols provided by their respective manufacturers. Malondialdehyde (MDA; Beyotime, China), total glutathione (GSH; Beyotime, China), and glutathione peroxidase activity levels (GPx; Beyotime, China) were measured according to provided instructions. Furthermore, Caspase-3 activity, Caspase-8 activity and Caspase-9 activity assays were conducted based on manufacturers’ guidelines from Beyotime Biotechnology Co., Ltd., China.

### 2.11 Mito-tracker immunofluorescent staining

According to the Mito-Tracker Deep Red FM (Beyotime, China) manual, cells were inoculated in a cell culture plate. Following treatment with drugs, the cell medium was removed. The prepared working solution of Mito-Tracker Deep Red FM was added and incubated at 37°C for 20 min. After staining, the cells were washed with PBS and fixed using fresh 3.7% formaldehyde for 15 min. Post-fixation, the cells underwent two washes with PBS before being incubated in 0.2% Triton X-100 in PBS at room temperature for 5 min, followed by another wash with PBS. A small volume of Hoechst 33342 staining solution was added and allowed to place at room temperature for 5 min. Subsequently, the slides were washed three times with PBS prior to sealing and observation under laser confocal microscopy.

### 2.12 Mitochondrial respiratory oxygen consumption rate (OCR) measurement

Mitochondrial respiration is assessed by oxygen consumption rate, which is conducted using a Seahorse XFe24 Analyzer (Agilent, Unites States) following the manufacturer’s instructions. The final results are analyzed utilizing Seahorse software.

### 2.13 Western blotting

Tissue or cells was lysed using RIPA buffer supplemented with a protease inhibitor cocktail (Roche, Unites States). Protein concentration was determined employing the Pierce BCA Protein Assay Kit (Beyotime, China). Low molecular weight markers (CST, Unites States) and protein samples were separated via sodium dodecyl sulfate-polyacrylamide gel electrophoresis (SDS-PAGE). The separated proteins were transferred onto a polyvinylidene fluoride membrane and blocked at room temperature for 1.5 h in Tris buffer containing 5% BSA. Membranes were incubated overnight at 4°C with antibodies against Bcl-2 (Abcam, UK; dilution: 1:1000), Bax (CST, Unites States; dilution: 1:1000), Caspase-3 (Affinity, China; dilution: 1:1000), Cleaved-Caspase-3 (CST, Unites States; dilution: 1:1000), Phospho-Drp-1(Ser616) (Affinity, China; dilution: 1:1000), CaMKII (Abmart, China; dilution: 1:1000), Phospho-CaMKⅡ Antibody (CST, Unites States; dilution: 1:1000), Cytochrome c (Biovision, Unites States; dilution: 1:200), and Glyceraldehyde-3-phosphate dehydrogenase (GAPDH) antibody (CST, Unites States; dilution: 1:5000). The secondary antibodies (CST, Unites States) utilized in this study are labeled with horseradish peroxidase and diluted at a ratio of 1:3000, followed by incubation at room temperature for 1.5 h. The immunoblot results were visualized using E-blot Plus. The relative band density of proteins in the protein immunoblot hybridization was normalized to GAPDH as an internal reference. By normalizing the data to the control value, the final results are expressed as fold changes.

### 2.14 Statistical analyses

Statistical analysis and plotting were conducted using GraphPad Prism version 8.0.2 (263) (GraphPad Software, Unites States) and SPSS version 23.0 (IBM Corporation, Unites States). Data are presented as mean ± SEM. One-way ANOVA and *post hoc* Dunnett’s tests were applied in the independent variable with more than two groups. A difference was deemed statistically significant when *p* < 0.05.

## 3 Results

### 3.1 DQ alleviates myocardial I/R injury *in vivo*

**FIGURE 1:**
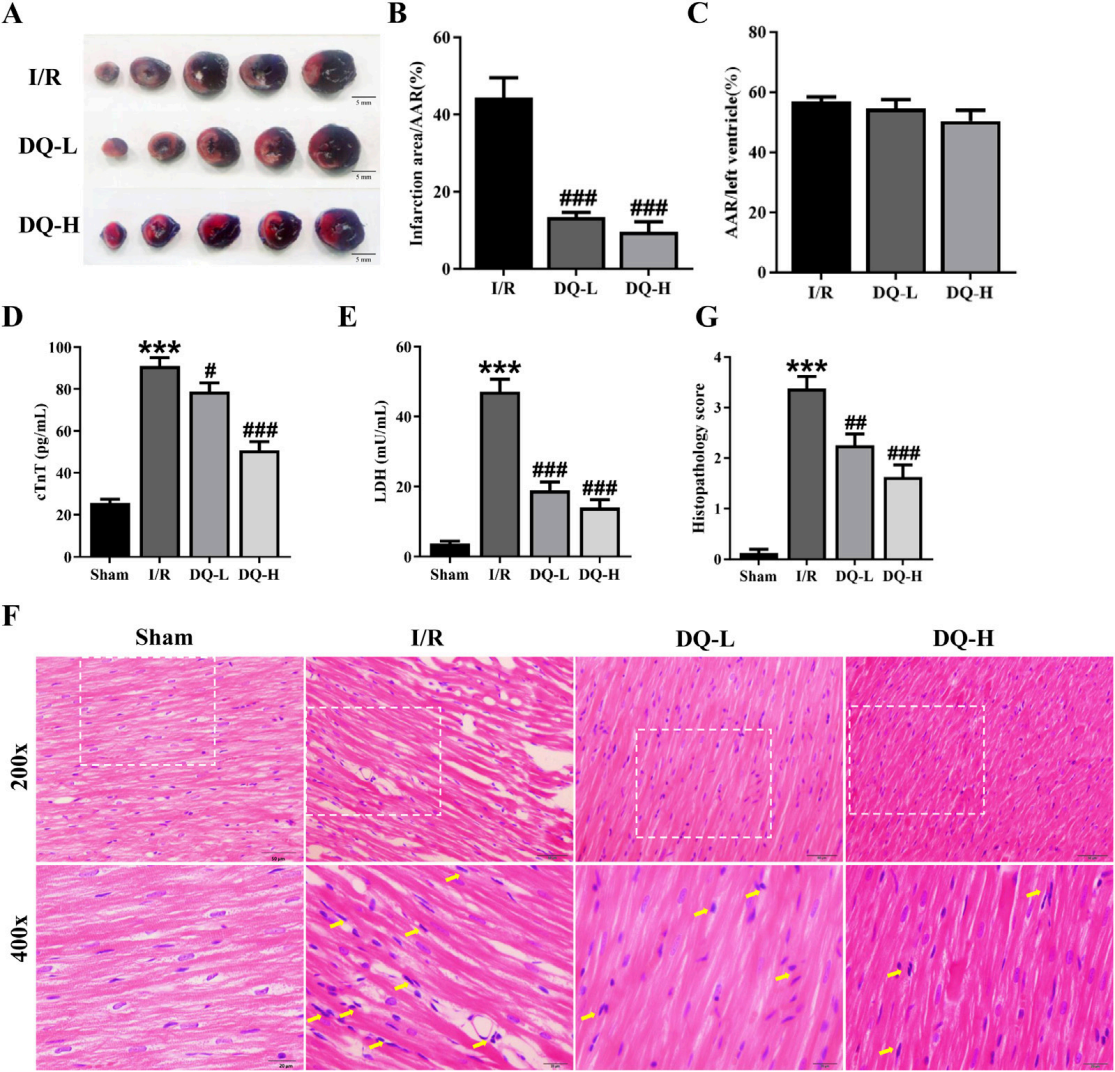
DQ mitigates myocardial I/R injury *in vivo*. **(A)** Representative heart slices stained with Evans blue/TTC double staining 24 h after I/R injury; Scale bar = 5 mm. The area at risk (AAR, Sum of white and red areas); healthy viable tissue (blue) and infarcted tissue (pale white). **(B, C)** Quantification of infarct size relative to AAR **(B)** and AAR relative to left ventricular mass **(C)**, n = 4 rats per group. **(D, E)** Serum levels of cTnT **(D)** and LDH **(E)** in rats, n = 6 rats per group. **(F)** Histopathological pictures of heart tissue sections stained with HE. The yellow arrows indicate typical inflammatory cells; Scale bar = 20 μm, 50 μm respectively, n = 4 rats per group. **(G)** Histopathology score, n = 4 rats with four randomly selected fields for each rat. ***p < 0.001 vs. Sham rats; ^#^ *p* < 0.05, ^##^ *p* < 0.01, ^###^ *p* < 0.001 vs. I/R group.

To assess the cardioprotective effect of DQ on I/R injury, we evaluated the infarct area through TTC-Evans blue staining (Figure 1A). Compared with I/R group, the ratios of infarct area to the area at risk (AAR) were significantly reduced in DQ-L and DQ-H groups (Figure 1B). The ratios of AAR to the total ventricular area (LV) in all groups had no significant difference (Figure 1C). Additionally, myocardial enzyme levels of cTnT and LDH in both DQ-L and DQ-H groups showed significant decreases compared with those observed in I/R rats (Figures 1D, E). We further examined alterations in heart tissue pathology via HE staining; results indicated that cardiomyocyte membranes appeared clear and intact within the Sham group, with muscle fibers arranged neatly without signs of inflammatory cell infiltration. Conversely, the myocardial fibers were irregularly arranged, the structure was disordered, extensive cardiomyocyte lysis, necrosis, and neutrophil infiltration were evident within the I/R group tissues; however, pretreatment with DQ significantly reversed these pathological changes (Figure 1F). DQ showed a significant improvement in myocardial histopathological lesions (Figure 1G). Compared with sham group, the histopathological scores of I/R group was significantly increased. Compared with the I/R group, the histopathological scores of DQ-L group, DQ-H group were significantly reduced. Collectively, these findings suggest that DQ effectively mitigates myocardial I/R injury *in vivo*.

### 3.2 DQ reduces cardiomyocyte apoptosis in the heart with I/R injury

**FIGURE 2:**
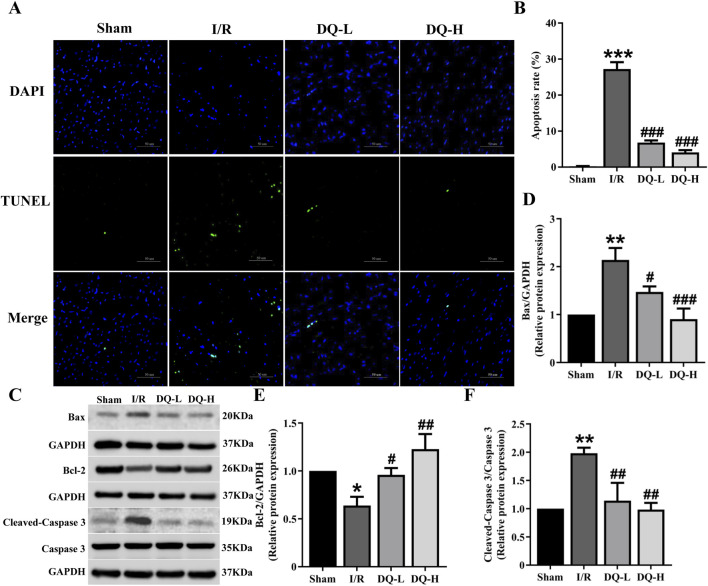
DQ reduces cardiomyocyte apoptosis in hearts subjected to myocardial I/R injury. **(A)** Apoptosis cells were assessed using TUNEL staining; The nucleus stained by DAPI was blue, and the positive apoptotic nucleus was green; Scale bar = 50 μm. **(B)** Quantitative analysis of apoptosis rate, n = 4 rats with eight randomly selected fields for each rat. **(C)** Representative images of Western blotting bands. **(D)** DQ decreased the protein expression level of Bax. **(E)** DQ increased the protein expression level of Bcl-2. **(F)** DQ reduced the protein expression level of Cleaved caspase-3. n = 4 rats per group, **(D–F)**, *p < 0.05, **p < 0.01, ***p < 0.001 vs. Sham group; ^#^ *p* < 0.05, ^##^ *p* < 0.01, ^###^ *p* < 0.001 vs. I/R group.

To investigate the role of DQ in mitigating cardiomyocyte apoptosis during myocardial I/R injury, we performed TUNEL staining to evaluate cardiomyocyte apoptosis in a rat model (Figure 2A). The I/R group exhibited a significant increase in the number of apoptotic cells compared to the Sham group (Figure 2B). Western blot analysis demonstrated that Bcl-2 protein levels were significantly reduced, while Bax protein levels were markedly elevated in the I/R group relative to those observed in the Sham group (Figures 2C–E). Treatment with DQ led to an upregulation of Bcl-2 expression and a downregulation of Bax expression (Figures 2C–E). Additionally, Cleaved-Caspase three levels were significantly higher in the I/R group compared to those found in the Sham group; however, these levels decreased following treatment with DQ (Figure 2F). Collectively, these findings suggest that DQ effectively reduces cardiomyocyte apoptosis associated with myocardial I/R injury.

### 3.3 DQ reduces mitochondrial damage in the heart with myocardial I/R injury

**FIGURE 3:**
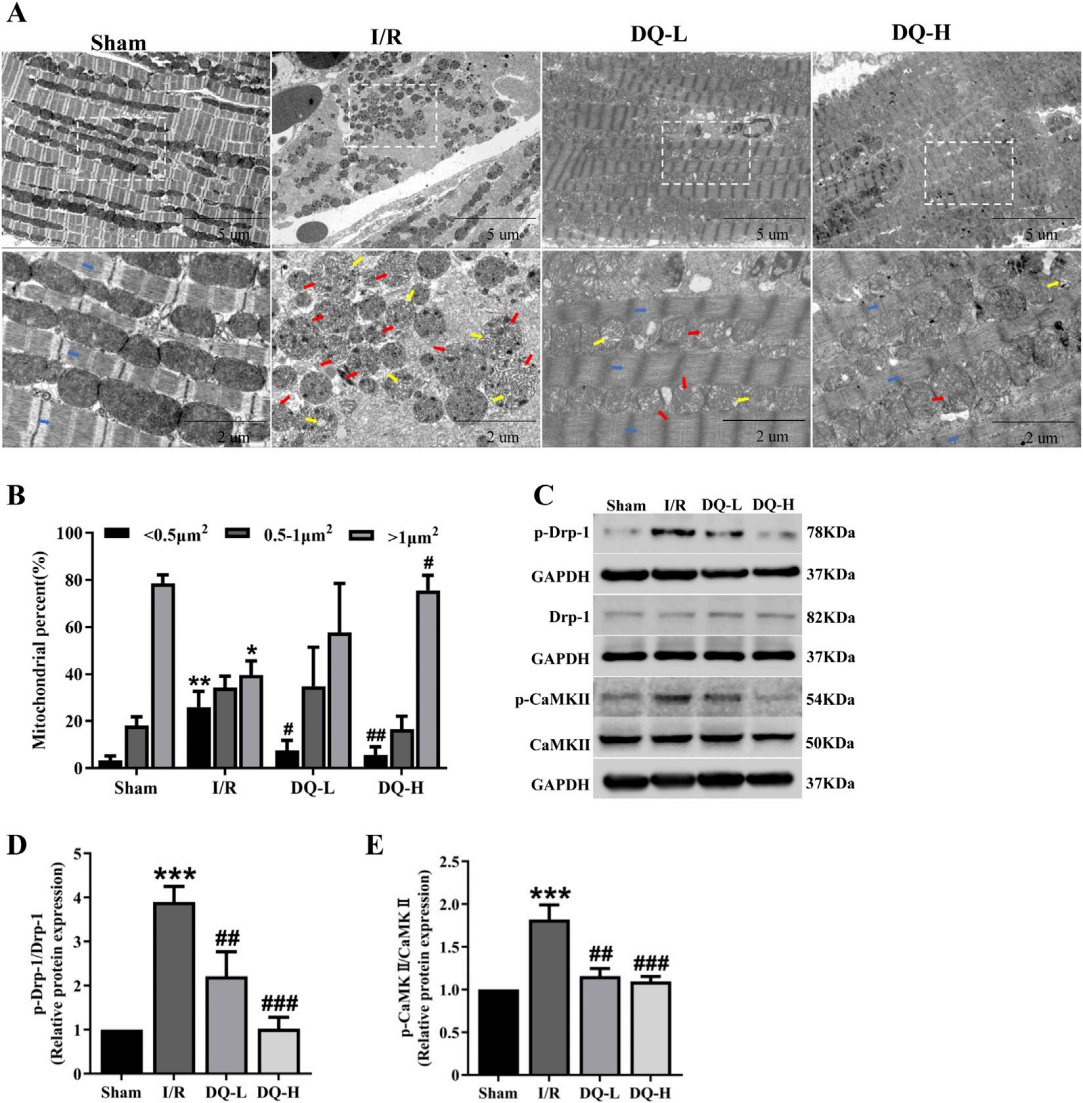
DQ ameliorates mitochondrial damage in the heart following myocardial I/R injury. **(A)** Representative micrographs of mitochondrial morphology were obtained using transmission electron microscopy. Typical mitochondrial cristae space was enlarged (red arrow); the cristae became short or dissolved (yellow arrow); and myofilaments (blue arrow) broke, dissolved and disappeared in I/R group. **(B)** Mitochondrial distribution based on individual area categorization by size (< 0.5, 0.5–1.0 or >1.0 μm^2^) in cardiomyocytes. **(C)** Representative images of Western blotting bands. **(D)** DQ reduced the protein expression of p-Drp-1. **(E)** DQ reduced the protein expression of p-CaMKⅡ. n = 4 rats per group, **(B, D, E)**. *p < 0.05, **p < 0.01, ***p < 0.001 vs. Sham group; #*p* < 0.05, ##*p* < 0.01, ###*p* < 0.001 vs. I/R group.

To investigate the protective mechanism by which DQ exerts its effects against I/R injury, we focused on mitochondrial dynamics. We examined morphological changes of mitochondria using transmission electron microscopy. As illustrated in Figure 3A, myofilaments within the Sham group exhibited neat and regular arrangements and mitochondrial morphology was normal. Conversely, within the I/R group, myofilaments broke, dissolved and disappeared. The mitochondrial cristae appeared irregularly shaped or fragmented. The cristae space was enlarged. In contrast, both DQ-L and DQ-H pretreatment groups demonstrated more regular myofilaments and cristae structure, accompanied by an increase in the number of larger mitochondria (Figure 3B). To further investigate the mechanism by which DQ regulates mitochondrial fission, we assessed the levels of fission-related proteins Drp-1 and its upstream regulator CaMKII using Western blotting (Figure 3C). The expression levels of p-Drp-1 and p-CaMKII in the I/R group were found to be elevated compared to those in the Sham group, while these levels significantly decreased following DQ treatment (Figures 3D, E). These findings suggest that DQ may mitigate mitochondrial damage by inhibiting Drp-1 phosphorylation and thereby reducing CaMKII activation to protect against myocardial I/R injury.

### 3.4 DQ reduces cardiomyocyte apoptosis induced by H_2_O_2_

**FIGURE 4:**
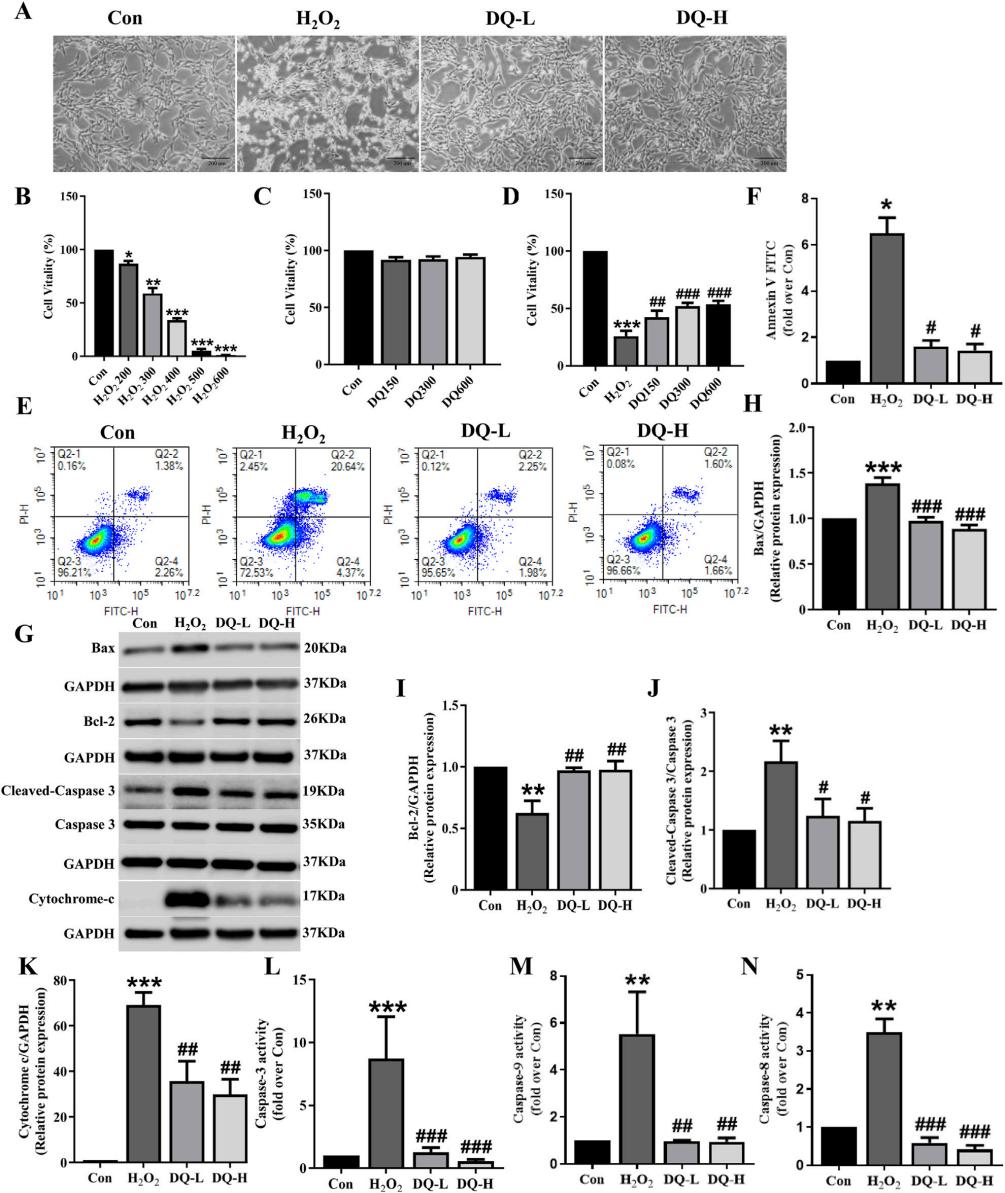
DQ mitigates H_2_O_2_-induced apoptosis in cardiomyocytes. **(A)** The morphology of H9c2 cells were observed under an inverted microscope (Scale bar = 200 μm). **(B–D)** Cell viability was assessed using MTT assay. n = 6 independent cell samples per group. **(E, F)** Flow cytometry was utilized to assess cell apoptosis rates, expressed as the sum of the percentages for early and late apoptotic stages, n = 3 independent cell samples per group. **(G)** Representative images of Western blotting bands. **(H)** DQ reduced the protein expression of Bax. **(I)** DQ increased the protein expression of Bcl-2. **(J)** DQ reduced the protein expression level of Cleaved caspase-3. **(K)** DQ reduced the protein expression level of Cytochrome C. n = 4 independent cell samples per group, **(H–K)**. **(L–N)** Test kit for detecting the release of Caspase3/9/8 enzyme activity in cells, n = 3 independent cell samples per group. *p < 0.05, **p < 0.01, ***p < 0.001 vs. Con group; ^#^ *p* < 0.05, ^##^ *p* < 0.01, ^###^ *p* < 0.001 vs. H_2_O_2_ group.

To investigate the protective effect of DQ on cardiomyocyte damage, we focused on the cellular apoptosis in a cell model of oxidative stress injury induced by H_2_O_2_ (Figure 4A). The MTT assay was employed to assay the survival rate of cardiomyocytes, revealing a gradual decrease in cell viability with increasing concentrations of H_2_O_2_ (Figure 4B). We selected 400 μM as the modeling concentration for subsequent experiments (Figure 4B). Furthermore, we treated H9c2 cells with different concentrations of DQ, and found that DQ (150–600 mg/mL) exhibited no cytotoxicity towards H9c2 cells (Figure 4C). Subsequently, we exposed H9c2 cells injured by H_2_O_2_ to different concentrations of DQ; results indicated that DQ significantly enhanced the viability of these injured cells (Figure 4D). The optimal concentrations were designated as the DQ-L group (300 mg/mL) and the DQ-H group (600 mg/mL, Figure 4D). Flow cytometry analysis revealed a significant increase in apoptosis rate within the H_2_O_2_ group compared to controls; conversely, both the DQ-L and DQ-H groups demonstrated markedly reduced apoptosis rates relative to the H_2_O_2_ group (Figures 4E, F). Additionally, Western blotting results indicated that exposure to H_2_O_2_ led to decreased expression levels of Bcl-2 and increased expression levels of Bax, Cleaved-Caspase-3, and Cytochrome C in cardiomyocytes when compared with control groups (Figures 4G–K). In contrast, treatment with DQ resulted in elevated Bcl-2 expression while decreasing Bax, Cleaved-Caspase-3, and Cytochrome C levels (Figures 4G–K). We also assessed enzyme activities for Caspase-3, Caspase-9, and Caspase-8 within these cells (Figures 4L–N). Compared to control groups, activities for all three caspases were significantly elevated in the H_2_O_2_-treated group; however, treatment with DQ notably reduced these enzymatic activities. In summary, our findings demonstrate that DQ effectively mitigates cardiomyocyte apoptosis induced by H_2_O_2_.

### 3.5 DQ reduces mitochondrial damage in cardiomyocytes induced by H_2_O_2_

**FIGURE 5:**
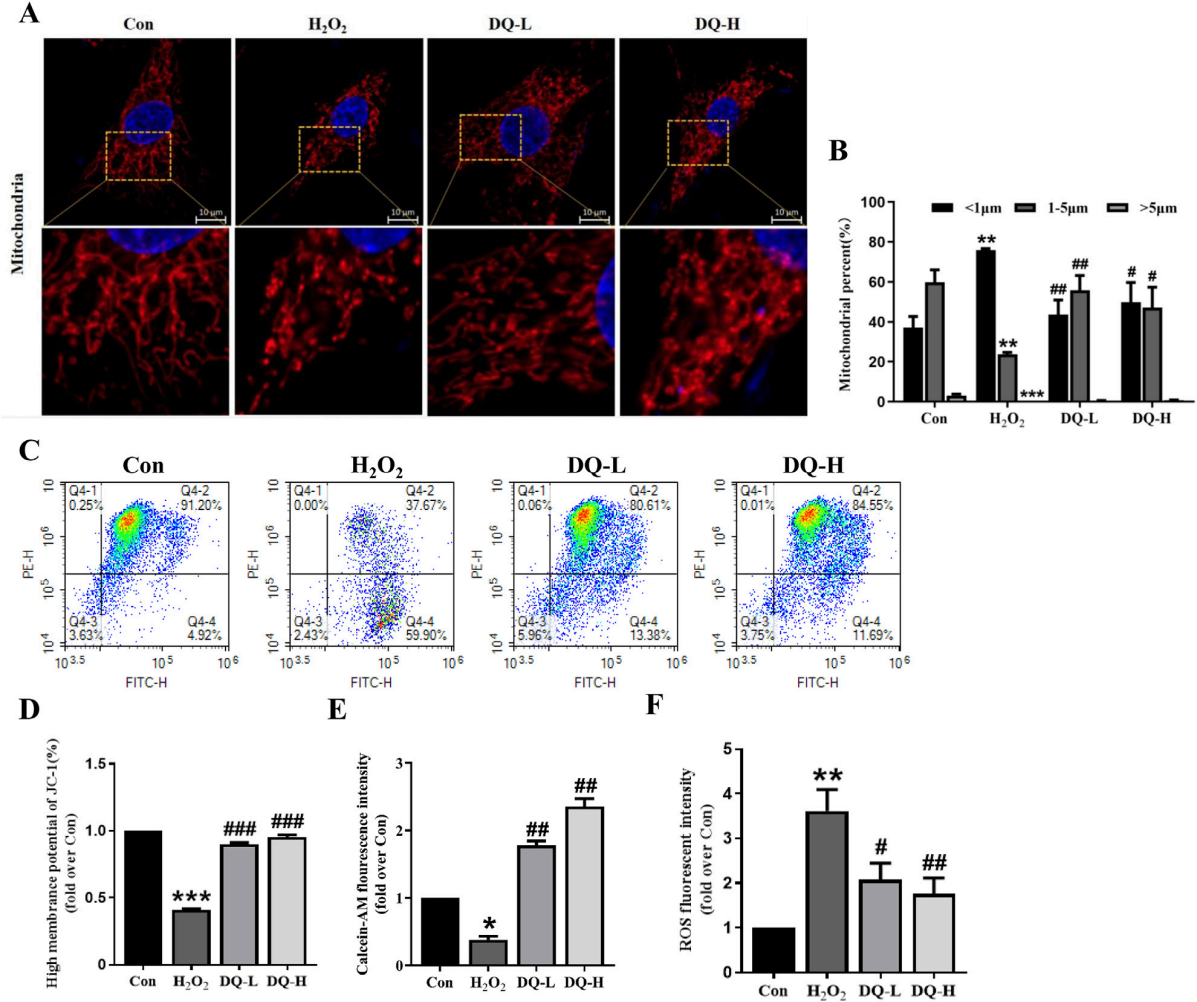
DQ mitigates mitochondrial damage in cardiomyocytes induced by H_2_O_2_. **(A)** Immunofluorescence staining to observe the mitochondria of cardiomyocytes (Scale bars = 10 μm). **(B)** Mitochondrial size distribution in cardiomyocytes, n = 4 independent cell samples per group. **(C, D)** DQ enhanced high membrane potential as measured by JC-1 assay. **(E)** DQ elevated calcein AM levels calculated by fluorescence intensity. **(F)** DQ reduced ROS levels quantified via fluorescence intensity. n = 3 independent cell samples per group, **(C–F)**. *p < 0.05, **p < 0.01, ***p < 0.001 vs. Con group; ^#^ *p* < 0.05, ^##^ *p* < 0.01, ^###^ *p* < 0.001 vs. H_2_O_2_ group.

To assess the protective effect of DQ on mitochondrial damage in cardiomyocytes caused by H_2_O_2_, we conducted mito-tracker immunofluorescent staining, JC-1 assays, mPTP analysis and ROS detection. The immunofluorescence staining revealed a distinct and rod-shaped mitochondrial grid structure was obvious and in the Con group (Figure 5A). In contrast, the H_2_O_2_ group exhibited significant mitochondrial damage characterized by the loss of grid structure and fragmentation. However, both the DQ-L and DQ-H groups effectively reversed these alterations; following DQ treatment, there was a notable reduction in punctate mitochondria alongside a significant increase in rod-shaped mitochondria (Figure 5B). Compared to the Con group, the membrane potential was significantly reduced in the H_2_O_2_ group; however, treatment with DQ-L and DQ-H effectively restored this parameter (Figures 5C, D). The fluorescence intensity of mitochondrial calcein AM was markedly decreased in the H_2_O_2_ group relative to the Con group, indicating an enhancement in mPTP opening following H_2_O_2_ treatment. Notably, pretreatment with DQ resulted in a significant increase in fluorescence intensity compared to the H_2_O_2_ group, suggesting that DQ inhibited H_2_O_2_-induced mPTP opening in H9c2 cells (Figure 5E). Furthermore, we measured intracellular ROS production and found that ROS release was markedly elevated in the H_2_O_2_ group compared to controls. Notably, ROS generation after treatment with either DQ-L or DQ-H was significantly lower than that observed in the H_2_O_2_ group (Figure 5F). Collectively, these data indicate that DQ can mitigate mitochondrial damage inflicted upon cardiomyocytes by H_2_O_2_.

### 3.6 DQ protects mitochondrial function in cardiomyocytes damaged by H_2_O_2_

**FIGURE 6:**
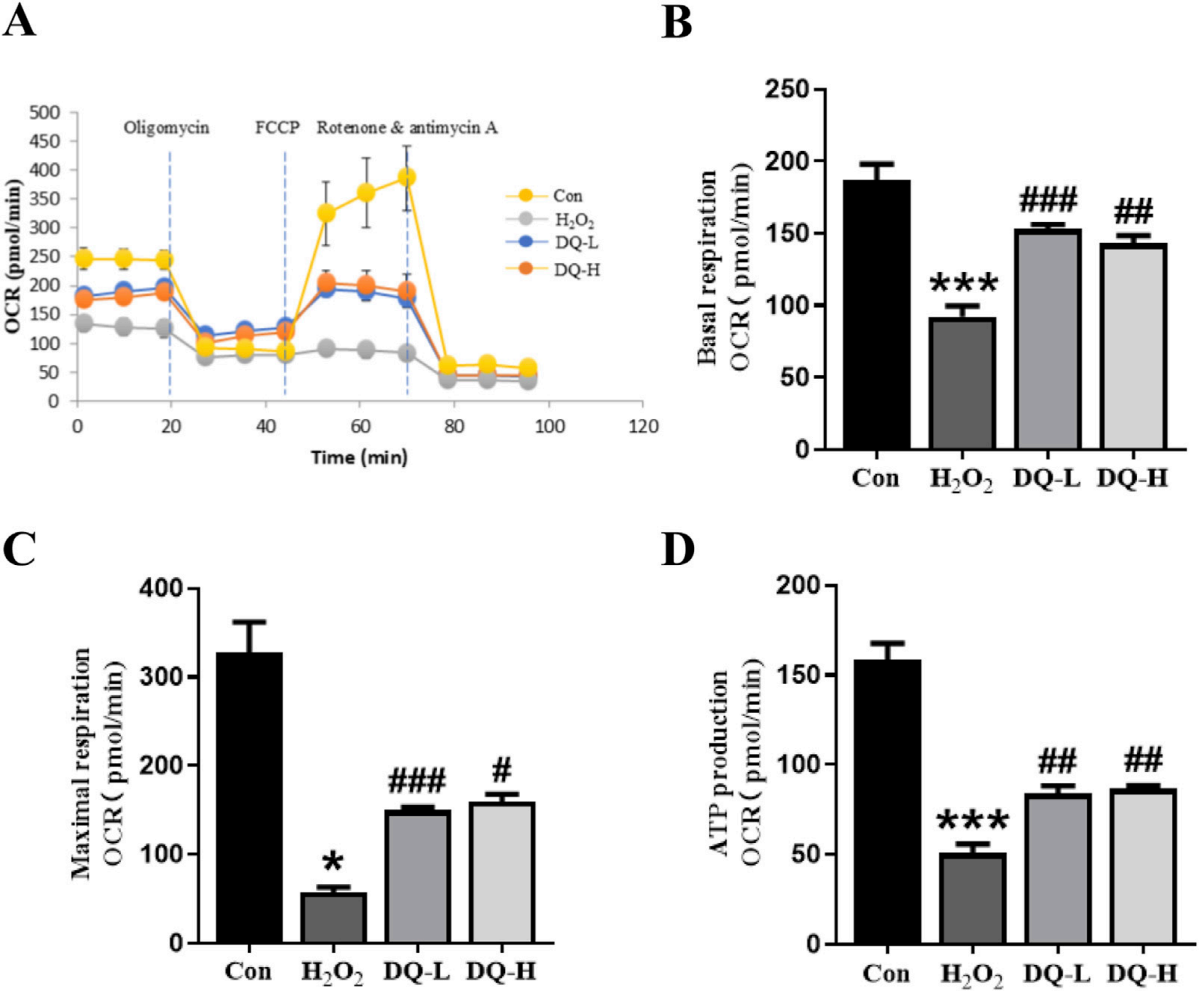
DQ protects mitochondrial function in cardiomyocytes *in vitro*. **(A)** OCR of mitochondrial respiration over time with sequential addition of oligomycin (1 μM), FCCP (2 μM), rotenone (0.5 μM) and antimycin (0.5 μM). **(B)** Basal respiration parameter. **(C)** Maximal respiration parameter. **(D)** ATP production parameter. n = 4 independent cell samples per group, **(A–D)**. *p < 0.05, ***p < 0.001 vs. Con group; ^#^ *p* < 0.05, ^##^ *p* < 0.01, ^###^ *p* < 0.001 vs. H_2_O_2_ group.

The oxygen consumption rates (OCRs) of H9c2 cells were assessed to evaluate mitochondrial function. A visual representation of real-time OCR measurements for evaluating mitochondrial function was obtained using the Seahorse XFe system (Figure 6A). Compared to the H_2_O_2_ group, both basal respiration OCR and maximal OCR were significantly enhanced in the DQ-treated groups, indicating that DQ treatment promotes higher respiratory efficiency under normal physiological conditions as well as stress conditions (Figures 6B, C). Additionally, mitochondrial ATP production within the DQ-treated groups also demonstrated a significant increase (Figure 6D).

### 3.7 DQ mitigates oxidative stress in cardiomyocytes induced by H_2_O_2_

**FIGURE 7:**
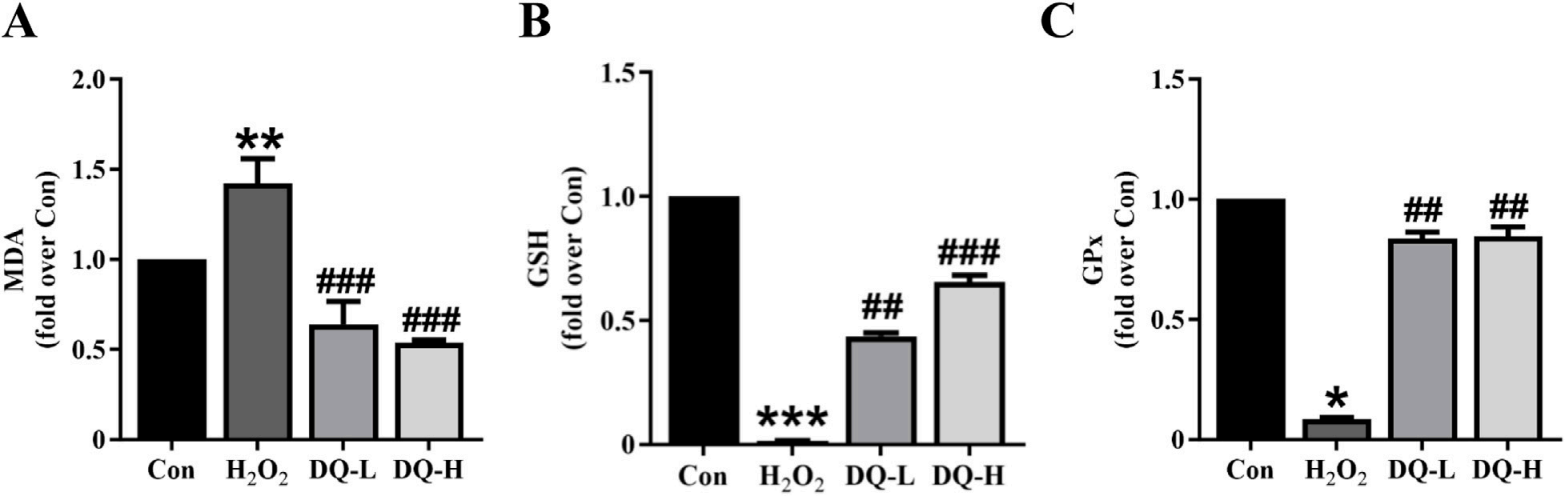
DQ alleviates oxidative stress in cardiomyocytes induced by H_2_O_2_. **(A)** Test kit for measuring the release of MDA enzyme activity within cells. **(B)** Test kit for assessing GSH enzyme activity release from cells. **(C)** Test kit for evaluating GPx enzyme activity release from cells. n = 3 independent cell samples per group, **(A–C)**. *p < 0.05, **p < 0.01, ***p < 0.001 vs. Con group; ^##^ *p* < 0.01, ^###^ *p* < 0.001 vs. H_2_O_2_ group.

Compared to the Con group, the MDA level in the H_2_O_2_ group was significantly elevated, and the GSH and GPx levels were markedly decreased. The DQ treatment effectively reversed these alterations (Figures 7A–C). These finding indicate that DQ can alleviate oxidative stress damage in cardiomyocytes caused by H_2_O_2_.

### 3.8 DQ reduces mitochondrial fission in cardiomyocytes induced by H_2_O_2_

**FIGURE 8:**
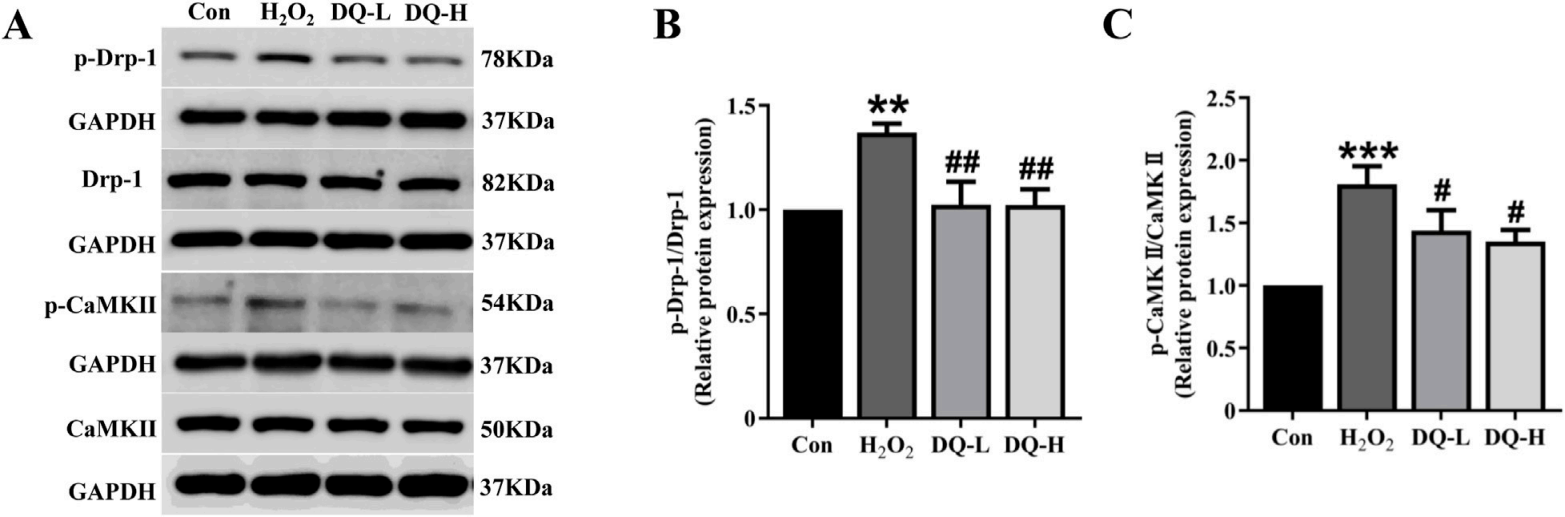
DQ reduces mitochondrial fission in cardiomyocytes induced by H_2_O_2_. **(A)** Representative images of Western blotting bands. **(B)** DQ decreased the protein expression level of p-Drp-1. **(C)** DQ decreased the protein expression level of p-CaMKII. n = 4 independent cell samples per group, **(B–C)**. **p < 0.01, ***p < 0.001 vs. Con group; ^#^ *p* < 0.05, ^##^ *p* < 0.01 vs. H_2_O_2_ group.

The expression levels of the mitochondrial fission protein Drp-1 and its upstream regulator CaMKII were assessed using Western blotting (Figure 8A). The results demonstrated that compared with the Con group, both p-Drp-1 (ser616) and p-CaMKⅡ levels were significantly increased in the H_2_O_2_ group; however, treatment with DQ group led to a reduction in the expression of p-Drp-1 and p-CaMKⅡ (Figures 8B, C). Based on these experiments, we conclude that DQ diminishes mitochondrial fission in cardiomyocytes caused by H_2_O_2_, thereby providing protective effects for these cells.

## 4 Discussion

**FIGURE 9:**
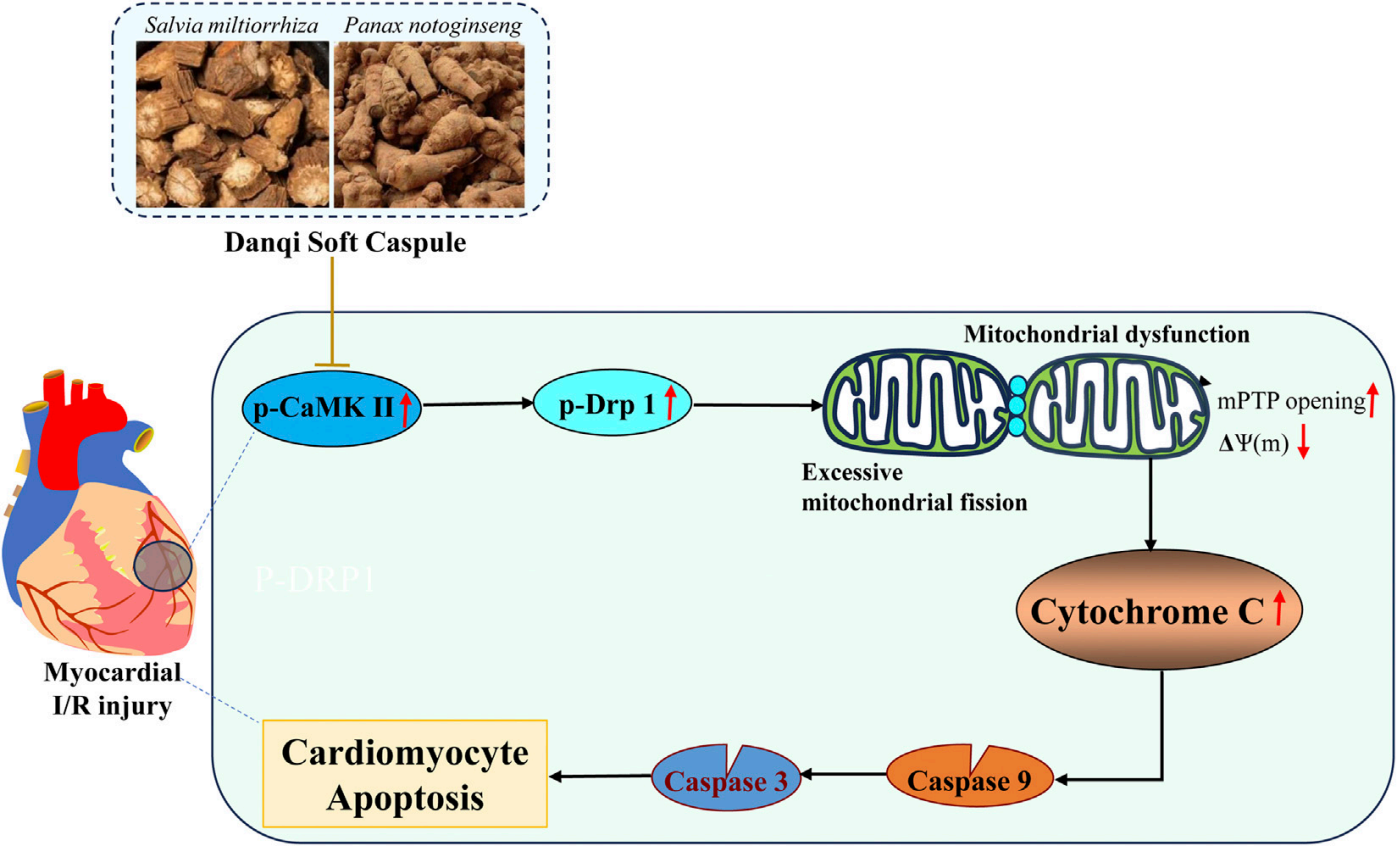
The putative mechanism underlying the therapeutic effect of DQ on myocardial I/R injury.

Chinese herbal compounds have demonstrated the ability to prevent I/R injury in various animal trials and clinical studies (Dong et al., 2023; Yang et al., 2023; Wu et al., 2024). In this study, we provide direct evidence supporting the protective effects of DQ on myocardial I/R injury. Firstly, DQ ameliorated myocardial damage and mitochondrial-mediated cardiomyocyte apoptosis in rats subjected to myocardial I/R injury. Subsequently, we confirmed that DQ could reduce oxidative stress levels and inhibit apoptosis by mitigating mitochondrial damage in H_2_O_2_-induced H9c2 cells. Finally, DQ preserved both the structure and function of mitochondria by reducing mitochondrial fission through inhibiting Drp-1 phosphorylation and reducing CaMKII activation *in vitro*. In summary, our findings substantiate that DQ protects against myocardial I/R injury and cardiomyocyte apoptosis through alleviating subsequent mitochondrial dysfunction and oxidative stress via inhibition of Drp-1 dependent mitochondrial fission. This provides experimental evidence for the efficacy of Chinese botanical drug in treating myocardial I/R injury (Figure 9).

Myocardial I/R injury represents a significant clinical challenge and is the primary contributor to the increasing incidence of post-ischemic heart failure worldwide. Despite its prevalence, there remains a lack of definitive therapeutic interventions (Liu et al., 2024). Traditional Chinese medicines have garnered considerable attention due to their enhanced therapeutic efficacy and fewer side-effects and the benefits of having multiple natural components, diverse targets, cost-effectiveness, and low toxicity profiles (Yang et al., 2023; Wu et al., 2024). The botanical drug pair consisting of *salvia miltiorrhiza* Bunge and *panax notoginseng (Burk.) F. H. Chen*, key constituents of DQ, is a classical formulation utilized for cardiovascular diseases in both China and Western countries (Zheng et al., 2013). This botanical drug Pair has demonstrated protective effects through mechanisms such as angiogenesis promotion, anti-inflammatory action, anti-thrombotic properties, and inhibition of retinal cell apoptosis (Zhang et al., 2022; Zeng et al., 2024). In the present study, an *in vivo* rat model of myocardial I/R injury was established following protocols outlined in previous research. Subsequent Evans blue/TTC staining revealed that DQ significantly inhibited myocardial infarct size compared to the I/R group. Additionally, serum levels of cardiac troponin T (cTnT) and lactate dehydrogenase (LDH) were evaluated within the I/R group. Notably, treatment with DQ resulted in a significant reduction in cTnT and LDH activities when compared with the I/R group. Consistently, we demonstrated that DQ increased cardiomyocyte viability in response to H_2_O_2_-induced oxidative stress injury *in vitro*. Based on these findings, we conclude that pretreatment with DQ can effectively reduce myocardial I/R injury *in vivo*.

Apoptosis is recognized as the primary form of cell death that mediates myocardial I/R injury (Chen et al., 2022). One of the principal pathways involved in apoptosis is oxidative stress and mitochondria-mediated activation of Caspase-3 (Wang et al., 2020; Gong et al., 2024). Oxidative stress induces the opening of the mitochondrial permeability transition pore (mPTP) and disrupts mitochondrial membrane potential, a phenomenon known as mitochondrial membrane potential depolarization. This disruption leads to subsequent release of Cytochrome c and activation of Caspase-3-dependent mitochondrial apoptosis (Steiner and Lang, 2017; Chistiakov et al., 2018; Bugger and Pfeil, 2020). Our findings indicate that DQ significantly reduced the Bax/Bcl-2 ratio and decreased cleaved-Caspase-3 expression in both myocardial I/R injury rats and H_2_O_2_-induced H9c2 cells, resulting in diminished cardiomyocyte apoptosis. *In vitro* studies demonstrated that DQ markedly inhibited reactive oxygen species (ROS), reduced mPTP opening, preserved mitochondrial membrane potential, and curtailed Cytochrome c release from mitochondria. Furthermore, DQ inhibited the activities of Caspase-3, Caspase-8, and Caspase-9. These observations underscore a definitive role for DQ in protecting against myocardial I/R injury by suppressing mitochondria-associated apoptosis. Additionally, we showed that DQ decreased MDA level—a product of lipid oxidation—and increased GSH as well as GPx, which are antioxidant enzymes in H9c2 cells subjected to H_2_O_2_ injury. Previous research has indicated that panax notoginseng saponin, one of the active components in DQ, exhibits anti-apoptotic effects by mitigating oxidative damage through signaling pathways related to oxidative stress and mitochondrial function (Chen et al., 2011; Zhou et al., 2018). Therefore, we conclude that DQ reduces cell apoptosis via its antioxidant properties along with its influence on mitochondria-mediated activation of the Caspase-3 pathway.

Mitochondrial damage is a critical pathological factor that contributes to myocardial I/R injury (Su et al., 2022). Mitochondria are the primary source of adenosine triphosphate (ATP) for energy-demanding cardiomyocytes, generating significant amounts of reactive oxygen species (ROS) and playing a crucial role in cardiomyocyte apoptosis during I/R injury (Pedriali et al., 2022; Huang and Zhou, 2023). Mitochondrial fission is recognized as an early alteration in mitochondrial morphology that occurs prior to mitochondrial dysfunction (Wang and Zhou, 2020). Evidence indicates that I/R injury promotes the phosphorylation of Drp-1 at Ser616, resulting in a rapid increase in the number of mitochondria fragments. This fragmentation is associated with elevated ROS production, diminished mitochondrial membrane potential, and activation of mitochondrial apoptosis (Wang and Zhou, 2020). On the other hand, myocardial I/R insults cause the elevation of intracellular Ca^2+^ (Andrienko et al., 2017) and ROS levels in cardiomyocytes, both of which lead to the activation of CaMKII (Adameova et al., 2022). CaMKII has been shown to upregulate proinflammatory cytokines, activate inflammation signaling cascades and results in mitochondrial dysfunction (Jiang et al., 2023), it plays a central role in cardiac I/R injury. Strategies aimed at negatively regulating mitochondrial fission by inhibiting Drp-1 or CaMKII in myocardial I/R injury models have demonstrated efficacy in preventing cardiac DNA damage, inflammation, and cardiac cell death (Ding et al., 2017; Yao et al., 2022). In our study, we observed an increase in mitochondrial fission within the oxidative stress injury cell model. DQ treatment resulted in decreased protein expression of phosphorylated Drp-1 and CaMKII while reducing both mitochondrial fragments and ROS generation in H9c2 cells exposed to H_2_O_2_. Additionally, DQ decreased MDA expression while increasing GSH and GPx levels in the H9c2 cells treated with H_2_O_2_. Collectively, these findings suggest that DQ mitigates mitochondrial fission by inhibiting Drp-1 phosphorylation and thereby reducing oxidative stress levels and CaMKII activation to protect against myocardial I/R injury and cell apoptosis.

In conclusion, our study demonstrates that DQ offers protection against myocardial I/R injury and cardiomyocyte apoptosis through its ability to reduce mitochondrial damage. Specifically, DQ inhibits cardiomyocyte mitochondrial fission and subsequent mitochondrial dysfunction. Targeting Drp-1 mediated mitochondrial fission and CaMKII activation may represent promising novel therapeutic strategies for addressing myocardial I/R injury. These results highlight the efficacy of DQ, a Chinese botanical drug, as a viable therapeutic intervention for treating myocardial I/R injury.

## Data Availability

The original contributions presented in the study are included in the article/Supplementary Material, further inquiries can be directed to the corresponding authors.
