# Corrigendum: Danqi soft caspule alleviates myocardial ischemia-reperfusion injury induced cardiomyocyte apoptosis by attenuating mitochondrial fission

**DOI:** 10.3389/fphar.2025.1625578

**Published:** 2025-06-05

**Authors:** Ye Yang, Cuiting Lin, Yan Wang, Yu Liu, Qiuxiong Chen, Shiyu Ma, Jin Ma

**Affiliations:** ^1^ The Second Clinical College of Guangzhou University of Chinese Medicine, The Second Affiliated Hospital of Guangzhou University of Chinese Medicine, Guangdong Provincial Hospital of Chinese Medicine, Guangzhou, China; ^2^ State Key Laboratory of Dampness Syndrome of Chinese Medicine, Guangzhou, China

**Keywords:** myocardial ischemia-reperfusion injury, oxidative stress, cell apoptosis, mitochondrial dynamics, Chinese botanical drug

In the published article, there was an error in Figures 1B,C as published. Upon re-examination with the guidance of our university professional statistician, we identified an error in the calculation of the LV area within the AAR/LV analysis. Our calculation method was based on the following definitions: the total area unstained by Evan’s blue (white area plus red area) represented the area at risk (AAR), and the blue area corresponded to the perfused tissue. The AAR/LV ratio was expressed as the percentage of AAR over total ventricular area. Regrettably, during the calculation of the LV area, the white infarct area was inadvertently double counted, which introduced inaccuracies into the statistical results. Upon conducting subsequent statistical analysis, we determined that there was no significant difference in the AAR/LV ratio, and the result is consistent with other articles. The corrected Figure 1 and its caption appear below.

**FIGURE 1:**
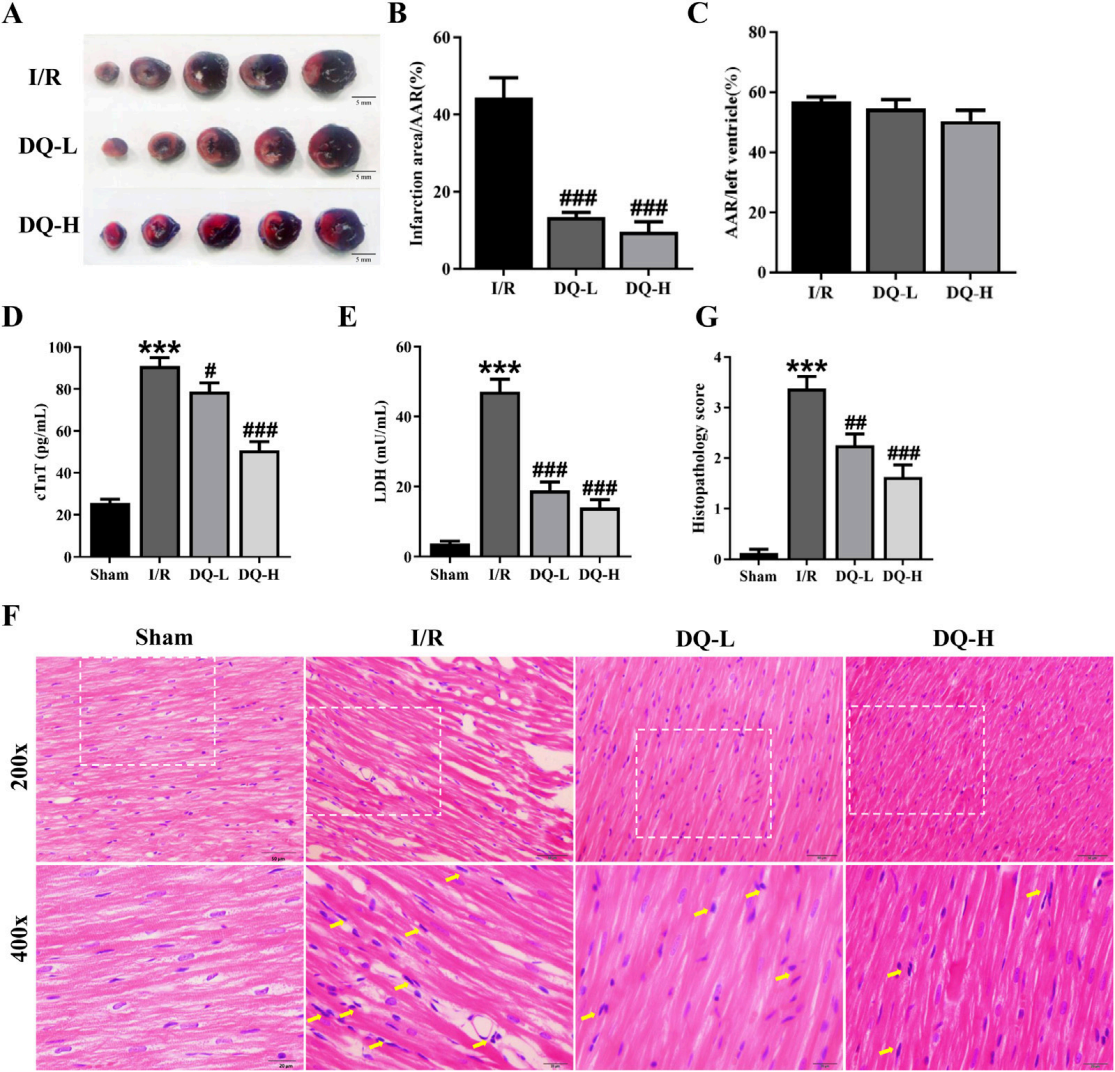
DQ mitigates myocardial I/R injury *in vivo*. **(A)** Representative heart slices stained with Evans blue/TTC double staining 24 h after I/R injury; Scale bar = 5 mm. The area at risk (AAR, Sum of white and red areas); healthy viable tissue (blue) and infarcted tissue (pale white). **(B,C)** Quantification of infarct size relative to AAR **(B)** and AAR relative to left ventricular mass **(C)**, n = 4 rats per group. **(D,E)** Serum levels of cTnT **(D)** and LDH **(E)** in rats, n = 6 rats per group. **(F)** Histopathological pictures of heart tissue sections stained with HE. The yellow arrows indicate typical inflammatory cells; Scale bar = 20 μm, 50 μm respectively, n = 4 rats per group. **(G)** Histopathology score, n = 4 rats with four randomly selected fields for each rat. ***p < 0.001 vs. Sham rats; ^#^ *p* < 0.05, ^##^ *p* < 0.01, ^###^ *p* < 0.001 vs. I/R group.

In the published article, there was an error due to inaccurate interpretation of results arising from statistical errors in the AAR analysis process.

A correction has been made to **3 Results**, *3.1 DQ alleviates myocardial I/R injury in vivo*, Paragraph Number 1. This sentence previously stated:

“The ratios of infarct area to ischemic risk area and ischemic area to the total ventricular volume in the I/R group were significantly increased compared with those in the Sham group; however, DQ treatment markedly reduced these ratios (Figures 1B,C).”

The corrected sentence appears below:

“Compared with I/R group, the ratios of infarct area to the area at risk (AAR) were significantly reduced in DQ-L and DQ-H groups (Figure 1B). The ratios of AAR to the total ventricular area (LV) in all groups had no significant difference (Figure 1C).”

The authors apologize for these errors and state that this does not change the scientific conclusions of the article in any way. The original article has been updated.

