# Supplementary material for: Danqi soft caspule alleviates myocardial ischemia-reperfusion injury induced cardiomyocyte apoptosis by attenuating mitochondrial fission
Source: Front Pharmacol. 2025 Mar 12;16:1526253. doi: 10.3389/fphar.2025.1526253 (PMC11936904; doi:10.3389/fphar.2025.1526253)
Supplement: Supplementary file 1 [file DataSheet1.docx]

**Danqi soft caspule alleviates myocardial ischemia-reperfusion injury induced cardiomyocyte apoptosis by attenuating mitochondrial fission**

Ye Yang^a,1^, Cuiting Lin^a,1^, Yan Wang^a^, Yu Liu^a^, Qiuxiong Chen^a,b^, Shiyu Ma^a,b, *^, Jin Ma^a,b, *^

**Supplementary file**

**Methods**

**Preparation of Danqi soft capsule (DQ)**

The DQ formulation consists of two traditional Chinese herbs, as detailed in Table S1. All herbs were sourced from authentic regional suppliers by China Meheco Great Wall Pharmaceutical Co., Ltd. (Beijing, China) and were identified using thin layer chromatography (TLC), following the guidelines outlined in Appendix VI B of the 2005 Edition of the Chinese Pharmacopoeia. The criteria for DQ pharmaceutical preparation (YBZ00332005-2007Z) were established by the China Food and Drug Administration. In brief, after drying, *Salvia miltiorrhiza* *Bunge* was macerated at room temperature with distilled water for 8 times (v/w), and decocted three times for one hour each time. The filtrates were combined and concentrated under conditions of -0.09 Mpa and 50°C to achieve a final concentration ranging from 1.35 to 1.4 g/ml (weight of original herb per volume of solution).. *Panax notoginseng (Burk.) F. H. Chen* was ground into fine powder and mixed with the concentrate obtained from *Salvia miltiorrhiza Bunge*. The mixture was then dried and pulverized again.

**Identification**

Put 2 g of DQ into a Soxhlet extractor and add 40 mL of petroleum ether (30-60℃). Reflux for 30 minutes, then discard the petroleum ether solution. Evaporate the petroleum ether from the residue, followed by adding 20 µL of methanol for ultrasonic treatment for 20 minutes. Filter the mixture, and use the filtrate as the test solution. Ginsenoside Rg1 and Notoginsenoside R1 were used as reference substances; methanol was added to prepare a mixed solution containing 1 mg per mL as the reference substance solution. Additionally, Sodium Danshensu was utilized as another reference substance; a solution containing 0.5 mg in 1 mL was prepared using 50% methanol. According to thin-layer chromatography testing (Chinese Pharmacopoeia, 2005 edition, Part I, Appendix VIB), fluorescent spots of identical color appeared at corresponding positions on the chromatogram of the reference substances.

**Ultra-high performance liquid chromatography (UPLC) fingerprint analysis and identification of DQ constituents**

A fingerprint method utilizing UPLC (ACQUITY UPLCTM, Waters Corp., USA) was developed for analyzing DQ constituents, complemented by combined UPLC/Q-TOF-MS/MS analysis using Bruker Daltonics equipment (Bremen, Germany). Specifically, an analysis was conducted on an 80% methanolic extract of DQ employing a BEH C18 column (2.1 mm × 50 mm; particle size: 1.7 μm). The mobile phase consisted of aqueous solutions containing 0.1% formic acid (A) and acetonitrile containing 1% formic acid (B), following this elution gradient: 5% B (0-3 min), 18%~23% B (3~13 min), 23%~40% B (13~15 min), 40%~70% B (18~18 min), and 70%~100% B (18~23 min). The flow rate was maintained at 250 μL/min, with detection wavelength set at 208 nm while keeping the column temperature constant at 30℃. An aliquot of 2µL of the test solution was injected into the UHPLC system. Subsequently, quadrupole-time of flight-mass spectrometry (Q-TOF-MS) was used for qualitative analysis under both positive and negative ion modes.

**Results**

**Chemical constituents of DQ**

Retention times, UV absorption data, and a chemical library were integrated to elucidate the chemical composition of DQ; A total of 24 chemical components were identified in the chromatogram of DQ (Figure S1, Table S2).

**TABLE S1** The information of Chinese medicines in DQ

| **Chinese**  **name** | **Latin name** | **Family** | **Place of Origin (Province)** | **Used part** | **Major compound in modern pharmacology study** |
| --- | --- | --- | --- | --- | --- |
| Sanqi | Panax notoginseng (Burk.) F. H. Chen | Araliaceae | Yunnan | rhizome | Notoginsenoside R1, Ginsenoside Rg1 |
| Danshen | Salvia miltiorrhiza Bunge | Lamiaceae | Jiangsu | rhizome | Salvianic acid A,  Salvianolic acid B |

Note: Panax notoginseng (Burk.) F. H. Chen and Salvia miltiorrhiza Bunge dosage ratio is 1:1.

**
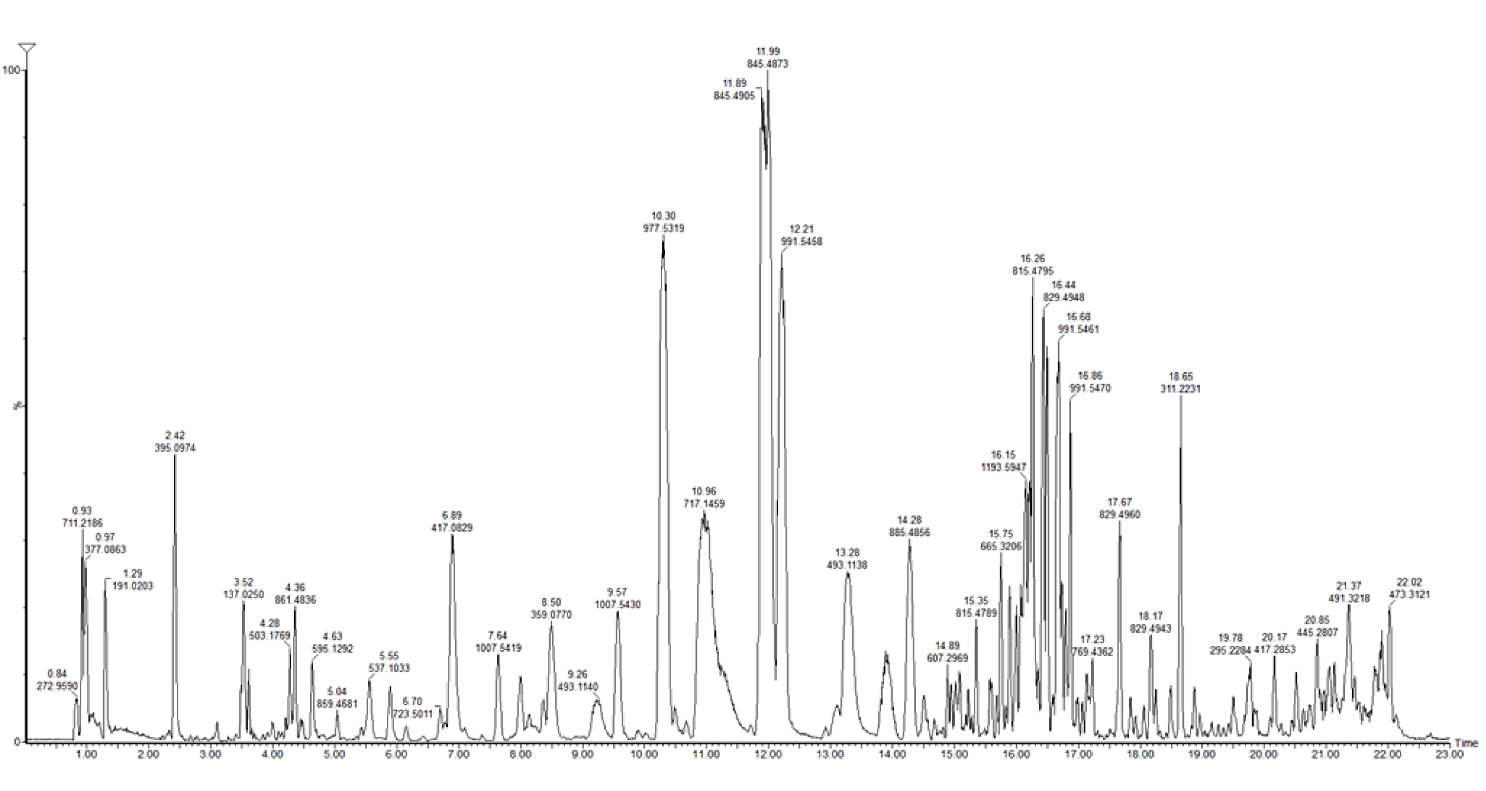
**

**FIGURE S1** Chromatograms of the isolated DQ compounds

**TABLE S2** Chemical constituents of DQ

| ***No.*** | **R*t* / min** | **Compound** | **Element composition** | **Source** |
| --- | --- | --- | --- | --- |
| 1 | 2.44 | Salvianic acid A | C9H10O5 | Salvia miltiorrhiza |
| 2 | 4.36 | Notoginsenoside H | C48H82O21 | Panax notoginseng |
| 3 | 4.67 | 10-Hydroxy-4,6-decadiynoic acid | C22H32O13 | Panax notoginseng |
| 4 | 5.55 | Lithospermic acid | C27H22O12 | Salvia miltiorrhiza |
| 5 | 7.64 | Notoginsenoside R3/Notoginsenoside R6 | C48H82O19 | Panax notoginseng |
| 6 | 9.57 | Notoginsenoside M/Notoginsenoside N | C48H82O19 | Panax notoginseng |
| 7 | 10.26 | Salvianolic acid B | C36H30O16 | Salvia miltiorrhiza |
| 8 | 10.30 | Notoginsenoside R1 | C47H80O18 | Panax notoginseng |
| 9 | 10.96 | Lithospermic acid B | C36H30O16 | Salvia miltiorrhiza |
| 10 | 11.99 | Ginsenoside Rg1 | C42H72O14 | Panax notoginseng |
| 11 | 12.21 | Ginsenoside Re | C48H82O18 | Panax notoginseng |
| 12 | 13.28 | Salvianolic acid A | C26H22O10 | Salvia miltiorrhiza |
| 13 | 15.35 | Notoginsenoside R2/Ginsenoside F5 | C41H70O13 | Panax notoginseng |
| 14 | 15.58 | Notoginsenoside M/Notoginsenoside N | C48H82O19 | Panax notoginseng |
| 15 | 16.01 | GinsenosideRf | C42H72O14 | Panax notoginseng |
| 16 | 16.06 | Ginsenoside Rb1 | C54H92O23 | Panax notoginseng |
| 17 | 16.26 | Ginsenoside R2 | C41H70O13 | Panax notoginseng |
| 18 | 16.44 | Ginsenoside F2 | C42H72O13 | Panax notoginseng |
| 19 | 16.48 | Ginsenoside Rh1 | C36H62O9 | Panax notoginseng |
| 20 | 16.68 | Ginsenoside Rd | C48H82O18 | Panax notoginseng |
| 21 | 16.87 | Ginsenoside Re | C48H82O18 | Panax notoginseng |
| 22 | 18.35 | Tanshindiol B | C18H16O5 | Panax notoginseng |
| 23 | 19.02 | Tanshinone IIA | C19H18O3 | Salvia miltiorrhiza |
| 24 | 20.59 | Tanshinone I | C18H12O3 | Salvia miltiorrhiza |
